# Differential gene expression analysis reveals pathways important in early post-traumatic osteoarthritis in an equine model

**DOI:** 10.1186/s12864-020-07228-z

**Published:** 2020-11-30

**Authors:** Annette M. McCoy, Ann M. Kemper, Mary K. Boyce, Murray P. Brown, Troy N. Trumble

**Affiliations:** 1 Department of Veterinary Clinical Medicine, University of Illinois Urbana-Champaign, 1008 W Hazelwood Dr, Urbana, IL USA; 2 Veterinary Population Medicine Department, University of Minnesota, 1365 Gortner Ave, St. Paul, MN USA; 3 Present Address: Crossroads Veterinary Clinic, Anderson, CA USA; 4 Department of Large Animal Clinical Sciences, University of Florida, 2015 SW 16th Avenue, Gainesville, FL USA

**Keywords:** Degenerative joint disease, Animal model, Synovium, Metacarpophalangeal joint, Osteochondral fragment, RNAseq

## Abstract

**Background:**

Post-traumatic osteoarthritis (PTOA) is a common and significant problem in equine athletes. It is a disease of the entire joint, with the synovium thought to be a key player in disease onset and progression due to its role in inflammation. The development of effective tools for early diagnosis and treatment of PTOA remains an elusive goal. Altered gene expression represents the earliest discernable disease-related change, and can provide valuable information about disease pathogenesis and identify potential therapeutic targets. However, there is limited work examining global gene expression changes in early disease. In this study, we quantified gene expression changes in the synovium of osteoarthritis-affected joints using an equine metacarpophalangeal joint (MCPJ) chip model of early PTOA. Synovial samples were collected arthroscopically from the MCPJ of 11 adult horses before (preOA) and after (OA) surgical induction of osteoarthritis and from sham-operated joints. After sequencing synovial RNA, Salmon was used to quasi-map reads and quantify transcript abundances. Differential expression analysis with the limma-trend method used a fold-change cutoff of log2(1.1). Functional annotation was performed with PANTHER at FDR < 0.05. Pathway and network analyses were performed in Reactome and STRING, respectively.

**Results:**

RNA was sequenced from 28 samples (6 preOA, 11 OA, 11 sham). “Sham” and “preOA” were not different and were grouped. Three hundred ninety-seven genes were upregulated and 365 downregulated in OA synovium compared to unaffected. Gene ontology (GO) terms related to extracellular matrix (ECM) organization, angiogenesis, and cell signaling were overrepresented. There were 17 enriched pathways, involved in ECM turnover, protein metabolism, and growth factor signaling. Network analysis revealed clusters of differentially expressed genes involved in ECM organization, endothelial regulation, and cellular metabolism.

**Conclusions:**

Enriched pathways and overrepresented GO terms reflected a state of high metabolic activity and tissue turnover in OA-affected tissue, suggesting that the synovium may retain the capacity to support healing and homeostasis in early disease. Limitations of this study include small sample size and capture of one point post-injury. Differentially expressed genes within key pathways may represent potential diagnostic markers or therapeutic targets for PTOA. Mechanistic validation of these findings is an important next step.

## Background

Osteoarthritis (OA) is a chronic, degenerative disease of joints that is characterized by pathology of the articular cartilage, subchondral bone, and synovium. Gross lesions typical of OA include cartilage fibrillation and erosions, subchondral bone sclerosis, and synovitis [1]. The complex interaction between these tissues and their relative contribution to the onset and progression of idiopathic OA is incompletely understood [1, 2]. However, in cases of post-traumatic osteoarthritis (PTOA), an inciting event (or events) is recognized that eventually leads to clinical, radiographic, and histologic signs of disease [3]. It is widely recognized that OA is the most common cause of chronic lameness in horses and places a significant burden on the equine industry due to the cost of treatment and loss of use of affected animals [4, 5]. The general population prevalence of OA in horses has been reported at 13.9% [6], although this markedly increases with age [7]. However, a study of Thoroughbred racehorses that died within 60 days of racing revealed that 33% had at least one full-thickness cartilage lesion in the metacarpophalangeal joint, and that severity of cartilage lesions strongly correlated with a musculoskeletal injury leading to death [5]. The majority of horses in this study were less than 3 years of age, emphasizing the importance of PTOA in young equine athletes. Despite the importance of this disease, the development of effective tools for its early diagnosis and treatment remains an elusive goal, in part due to a lack of knowledge regarding the early progression of PTOA.

Altered gene expression represents the earliest discernable disease-related change, measurable before biochemical markers of cartilage degradation, radiographic changes, etc., and can provide valuable information about disease pathogenesis as well as identify potential therapeutic targets. However, to date, the majority of studies examining global gene expression changes in osteoarthritic joints compared to normal have used end-stage diseased tissue [8–11], and therefore it is not known if any of the reported differentially expressed genes also play a role in early disease.

While cartilage lesions are traditionally considered the hallmark of OA, the synovium is known to be a key player in disease development and progression via its role in inflammation [3, 12, 13]. In fact, evidence suggests that pro-inflammatory mediators released by synoviocytes may be both a cause and consequence of cartilage damage in OA, making the synovium a viable target for therapeutic interventions [12]. Synovium also offers an attractive option for tissue-based diagnostic testing as it is easily collected via arthroscopy with minimal donor site morbidity. However, although histological changes have been described in experimentally-induced [14] and naturally-occurring [12] OA, little is known about how OA alters gene expression in synovium. Existing reports of gene expression changes in synovium at the time of surgical intervention after an injury have specifically focused on inflammatory mediators [15], but it is likely that genes involved in pathways other than inflammation also play an important role in PTOA. The aim of this work was to establish an accurate profile of the earliest molecular events that occur in the joint after the induction of PTOA in an experimental equine model. This non-terminal metacarpophalangeal joint (MCPJ) chip model results in mild, measurable morphological and histological changes, and was specifically designed to recapitulate the early stages of PTOA [16].

## Results

### Sequencing and clustering by multi-dimensional scaling (MDS)

**Fig. 1:**
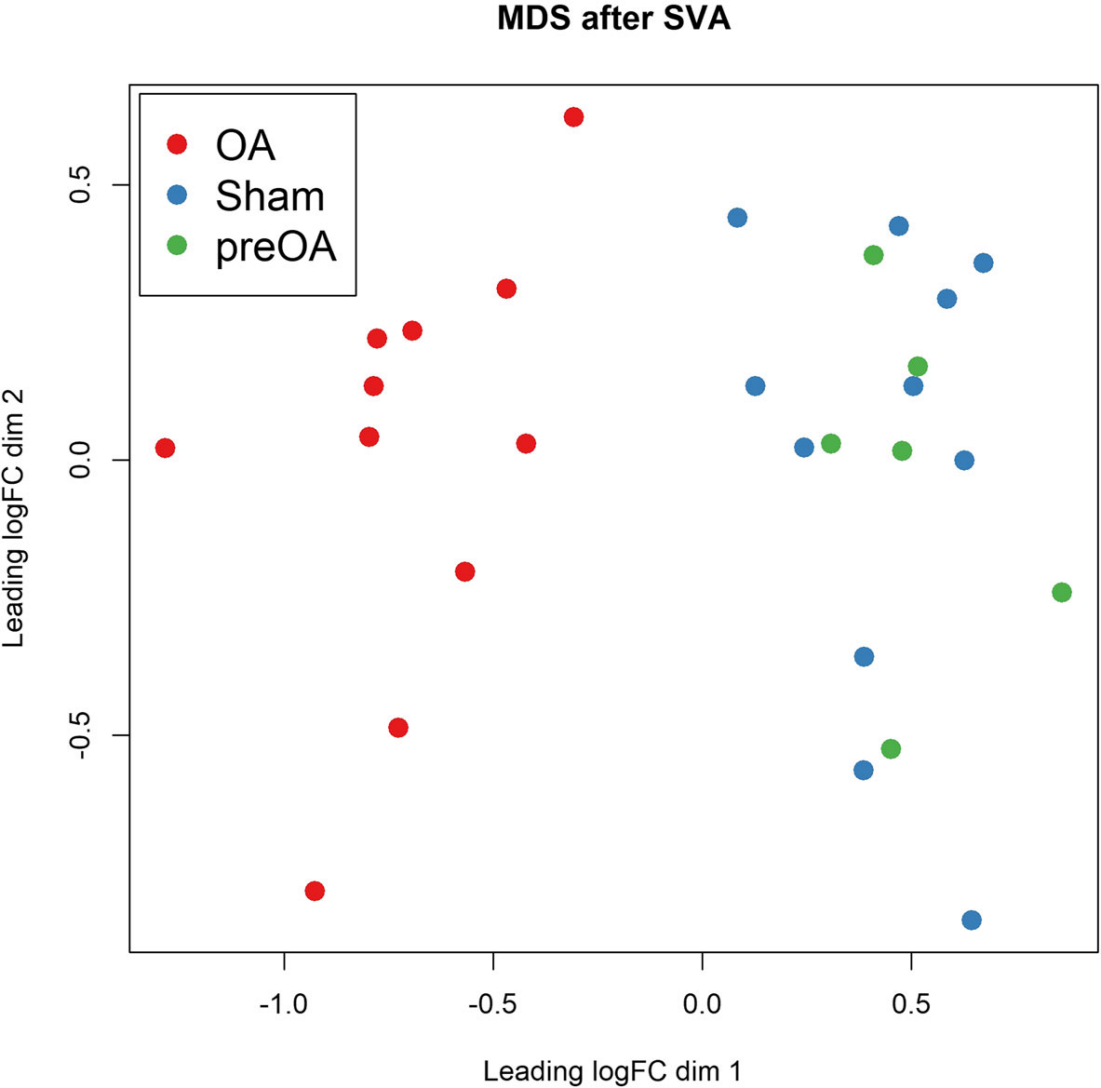
Multi-dimensional scaling (MDS) plot showing clustering of samples based on normalized gene expression values (logCPM), with surrogate variables (SVA) removed. OA = osteoarthritis samples, red; Sham = sham samples, blue, preOA = samples prior to induction of osteoarthritis, green. Sham and preOA samples were combined for downstream analyses

RNA of sufficient quantity and quality for sequencing was extracted from 28 banked synovial samples (6 preOA, 11 OA, 11 sham). Sequencing yielded 15.7–29.4 million paired-end reads per sample (Additional file 1). A total of 13,880 genes were expressed across all samples. After removal of surrogate variables, MDS showed stratification on Dimension 1 between the OA samples and the preOA and sham samples (Fig. 1). Sham and preOA samples did not cluster separately and these data were combined for all downstream analyses.

### Differential expression (DE)

**Fig. 2:**
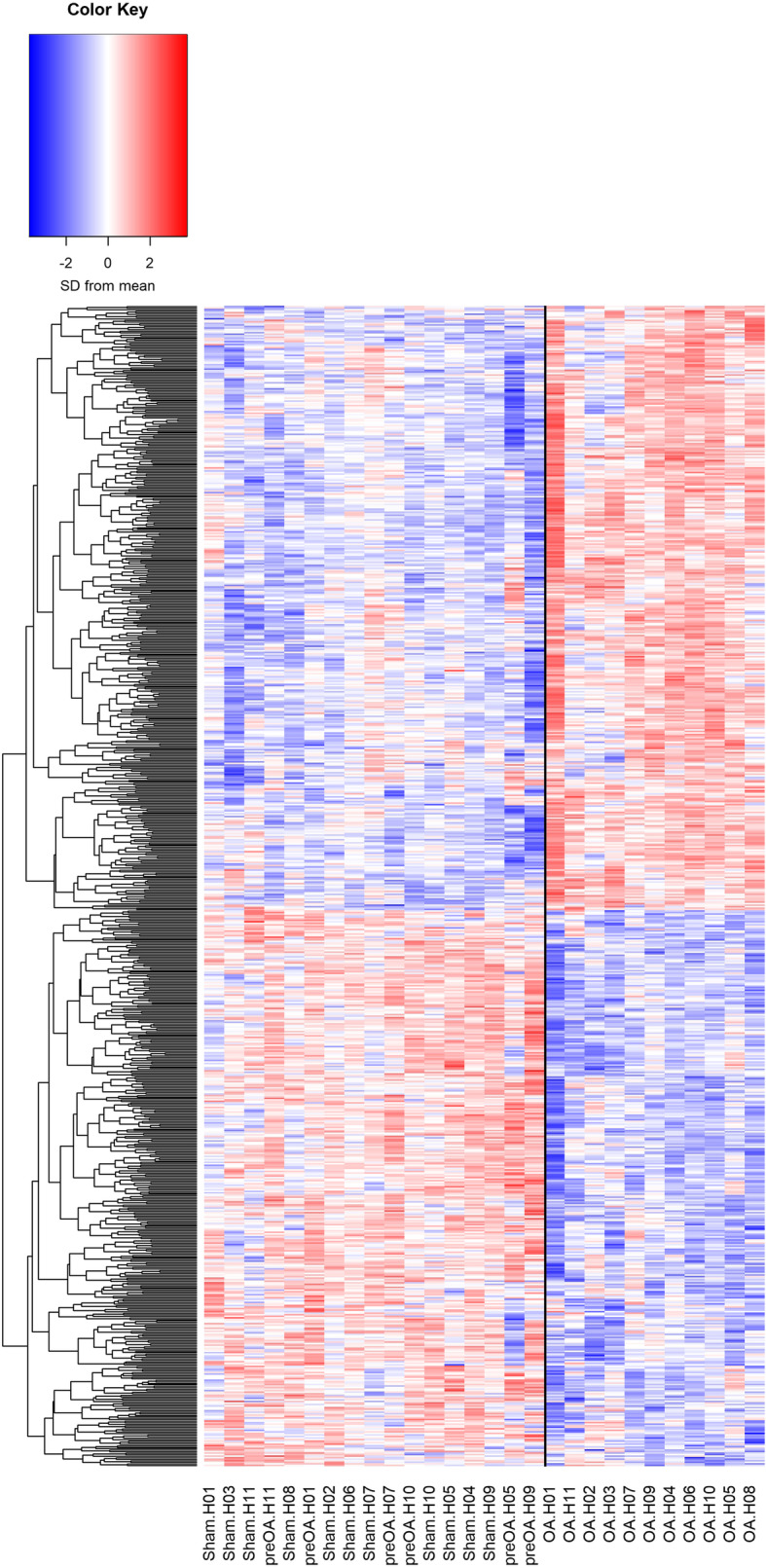
Heatmap of 762 DE genes in 17 non-affected (combined sham and preOA) and 11 OA samples. A complete list of DE genes can be found in Additional file 2

Initial DE analysis did not reveal any differences between the preOA and sham samples; therefore, these were combined into a single group, designated “non-affected”, and compared to gene expression in the OA samples. There were 762 genes DE (fold-change [FC] > |1.1|, FDR < 0.05) between OA and non-affected samples (Fig. 2, Additional file 2). Of these, 397 genes were upregulated in the OA samples, while 365 were downregulated in the OA samples.

### Functional annotation and pathway/network analysis

PANTHER reports a hierarchal organization of Gene ontology (GO)-Slim terms overrepresented when comparing OA to non-affected samples. GO terms could be assigned to 676 of the 762 DE genes. Among these, there were a total of 56 overrepresented GO terms: 29 Biological Process terms falling within eight hierarchical categories, 6 Molecular Function terms falling within three hierarchical categories, and 21 Cellular Component terms falling within eight hierarchical categories. The terminal hierarchical overrepresented terms are shown in Table 1.

**Table 1:** Overrepresented GO-Slim terms among DE genes

	Genes in reference list	Genes in analyzed list	Fold enrichment	Raw ***p***-value	FDR
**GO-Slim Biological Process**					
Angiogenesis (GO:0001525)	30	8	8.28	1.84E-05	0.0025
Extracellular matrix organization (GO:0030198)	69	15	6.75	4.45E-08	2.66E-05
Cell morphogenesis (GO:0000902)	60	9	4.66	2.82E-04	0.023
Regulation of cell migration (GO:0030334)	70	9	3.99	7.79E-04	0.047
Regulation of cAMP-mediated signaling (GO:0043949)	118	12	3.16	7.76E-04	0.048
Transmembrane receptor protein tyrosine kinase signaling pathway (GO:0007169)	197	18	2.84	1.48E-04	0.013
Cell adhesion (GO:0007155)	373	34	2.83	2.15E-07	6.43E-05
Actin cytoskeletal organization (GO:0030036)	179	16	2.78	4.26E-04	0.027
**GO-Slim Molecular Function**					
Extracellular matrix structural constituent (GO:0005201)	53	12	7.03	6.82E-07	0.0002
Metalloendopeptidase activity (GO:0004222)	42	7	5.18	7.66E-04	0.043
Actin binding (GO:0003779)	148	20	4.20	3.11E-07	0.0002
**GO-Slim Cellular Component**					
Collagen trimer (GO:0005581)	10	4	12.42	7.32E-04	0.015
Apical junction complex (GO:0043296)	11	4	11.29	9.73E-04	0.018
Collagen-containing extracellular matrix (GO:0062023)	33	10	9.41	6.16E-07	6.91E-05
Dendritic spine (GO:0043197)	27	5	5.75	2.91E-03	0.048
Supramolecular fiber (GO:0099512)	54	9	5.18	1.39E-04	0.0046
Actin cytoskeleton (GO:0015629)	225	25	3.45	3.28E-07	4.90E-05
Receptor complex (GO:0043235)	177	16	2.81	3.80E-04	0.0085
Integral component of plasma membrane (GO:0005887)	733	51	2.16	8.91E-07	7.99E-05

The reference list is *Homo sapiens* UniProt IDs and included 20,996 genes; the analyzed list is comprised of the UniProt IDs for 676 DE genes. *FDR* False discovery rate (significance set at 0.05). A complete hierarchical list of overrepresented terms can be found in Additional file 3. These data are represented in graphical form in Additional file 4

**Fig. 3:**
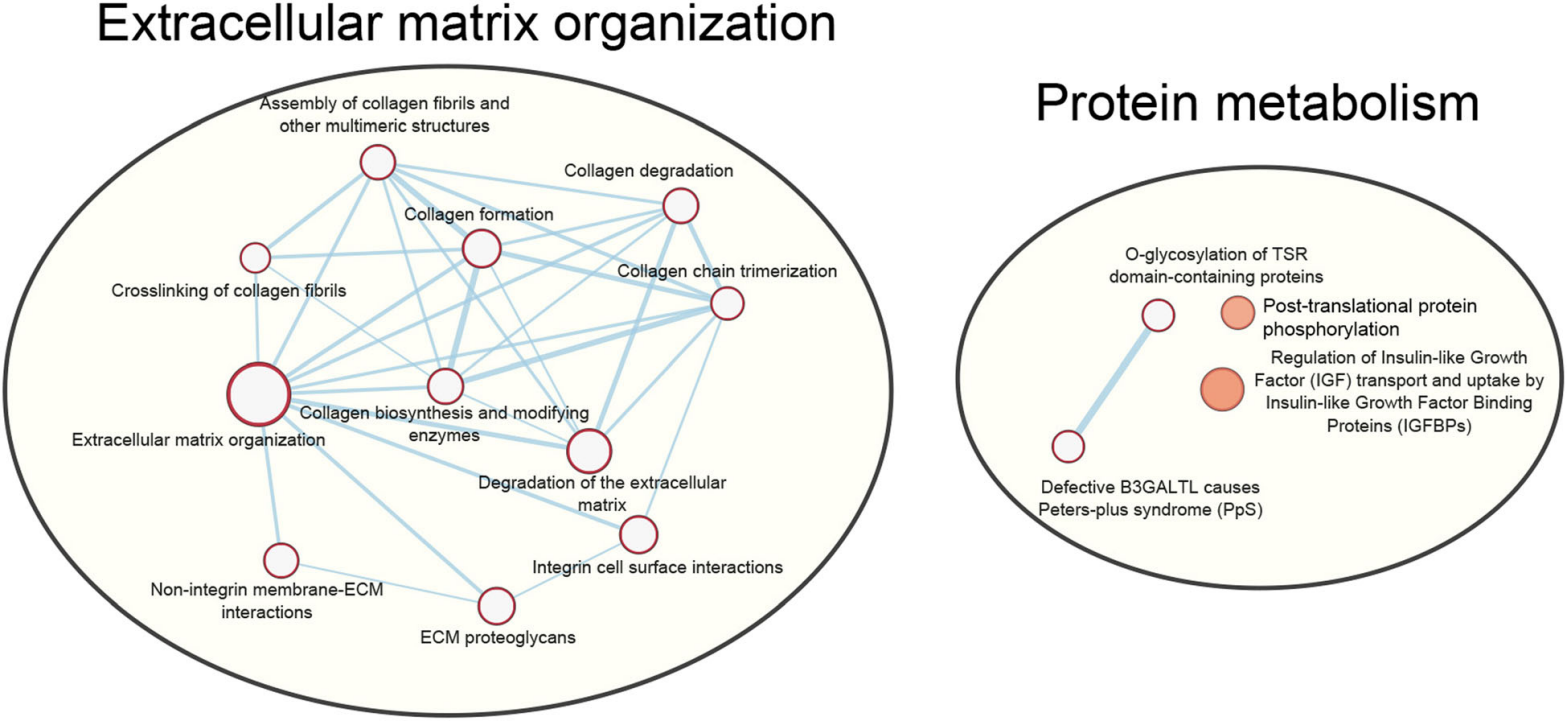
Networks of enriched pathways for extracellular matrix organization and protein metabolism identified by Reactome among DE genes. A complete list genes within these pathways can be found in Additional file 5

Reactome assigned 450 of the 762 DE genes to 1360 pathways, of which 17 reached the designated level of statistical significance (false discovery rate [FDR] < 0.05) (Table 2). Eleven of these enriched pathways fell generally into the category of extracellular matrix (ECM) organization (“ECM organization”, “degradation of the ECM”, “collagen degradation”, “ECM proteoglycans”, “assembly of collagen fibrils and other multimeric structures”, “collagen formation”, “integrin cell surface interactions”, “collagen biosynthesis and modifying enzymes”, “collagen chain trimerization”, “non-integrin membrane-ECM interactions”, “crosslinking of collagen fibrils”), while four were related to protein metabolism (“O-glycosylation of TSR domain-containing proteins”, “defective B3GALTL causes Peters-plus syndrome”, “regulation of insulin-like growth factor transport and uptake by insulin-like growth factor binding proteins”, “post-translational protein phosphorylation”) (Fig. 3). For all pathways falling in these two broad categories, more than 75% of the DE genes were upregulated in the OA samples when compared to the non-affected samples (Additional file 5). Enriched pathways relevant to ECM organization were largely driven by upregulation of numerous collagen sub-types and small extracellular matrix proteins in the OA samples, as well as several matrix metalloproteinases (MMPs) and ADAMTS (a disintegrin and metalloproteinase with thrombospondin motif) protein family members. MMPs, ADAMTS proteins, and growth factors/growth factor receptors were prominent drivers of the protein metabolism pathways. There was substantial overlap of genes between enriched pathways. In fact, of the 84 unique DE genes assigned to the 17 significantly enriched pathways, only ten were found only in a single pathway. Notably, while most DE genes in the enriched pathways had a 1.5- to 3-fold expression change between groups, MMP1, MMP9, and MMP13 exhibited much greater upregulation in the OA samples, with 8.5-fold, 7-fold, and 12.7-fold increases in expression, respectively.

**Table 2:** Enriched pathways identified by Reactome among DE genes

Pathway	Genes in reference list	Genes in analyzed list	***P***-value	FDR
ECM organization (R-HSA-1474244)	329	63	2.70E-12	3.98E-09
Degradation of the ECM (R-HSA-1474228)	148	33	1.42E-08	1.05E-05
Collagen degradation (R-HSA-1442490)	69	21	4.32E-08	2.12E-05
ECM proteoglycans (R-HSA-3000178)	79	21	3.86E-07	1.42E-04
Assembly of collagen fibrils and other multimeric structures (R-HSA-2022090)	67	19	5.32E-07	1.56E-04
Collagen formation (R-HSA-1474290)	104	24	7.12E-07	1.74E-04
Integrin cell surface interactions (R-HSA-216083)	86	21	1.46E-06	3.06E-04
Collagen biosynthesis and modifying enzymes (R-HSA-1650814)	76	19	3.25E-06	5.99E-04
Collagen chain trimerization (R-HSA-8948216)	44	14	4.50E-06	7.34E-04
O-glycosylation of TSR domain-containing proteins (R-HSA-5173214)	41	13	1.01E-05	0.001
Non-integrin membrane-ECM interactions (R-HSA-3000171)	61	16	1.06E-05	0.001
Crosslinking of collagen fibrils (R-HSA-2243919)	24	9	6.58E-05	0.008
Defective B3GALTL causes Peters-plus syndrome (R-HSA-5083635)	39	11	1.32E-04	0.015
Regulation of Insulin-like Growth Factor transport and uptake by Insulin-like Growth Factor binding proteins (R-HSA-381426)	127	22	1.43E-04	0.015
Post-translational protein phosphorylation (R-HSA-8957275)	109	19	3.66E-04	0.036
Phospholipase C-mediated cascade; FGFR2 (R-HSA-5654221)	25	8	4.71E-04	0.043
RUNX2 regulates genes involved in cell migration (R-HSA-8941332)	14	6	5.40E-04	0.046

*FDR* False discovery rate (significance set at 0.05), *ECM* Extracellular matrix, *R-HSA-XXX* Reactome pathway identifiers

**Fig. 4:**
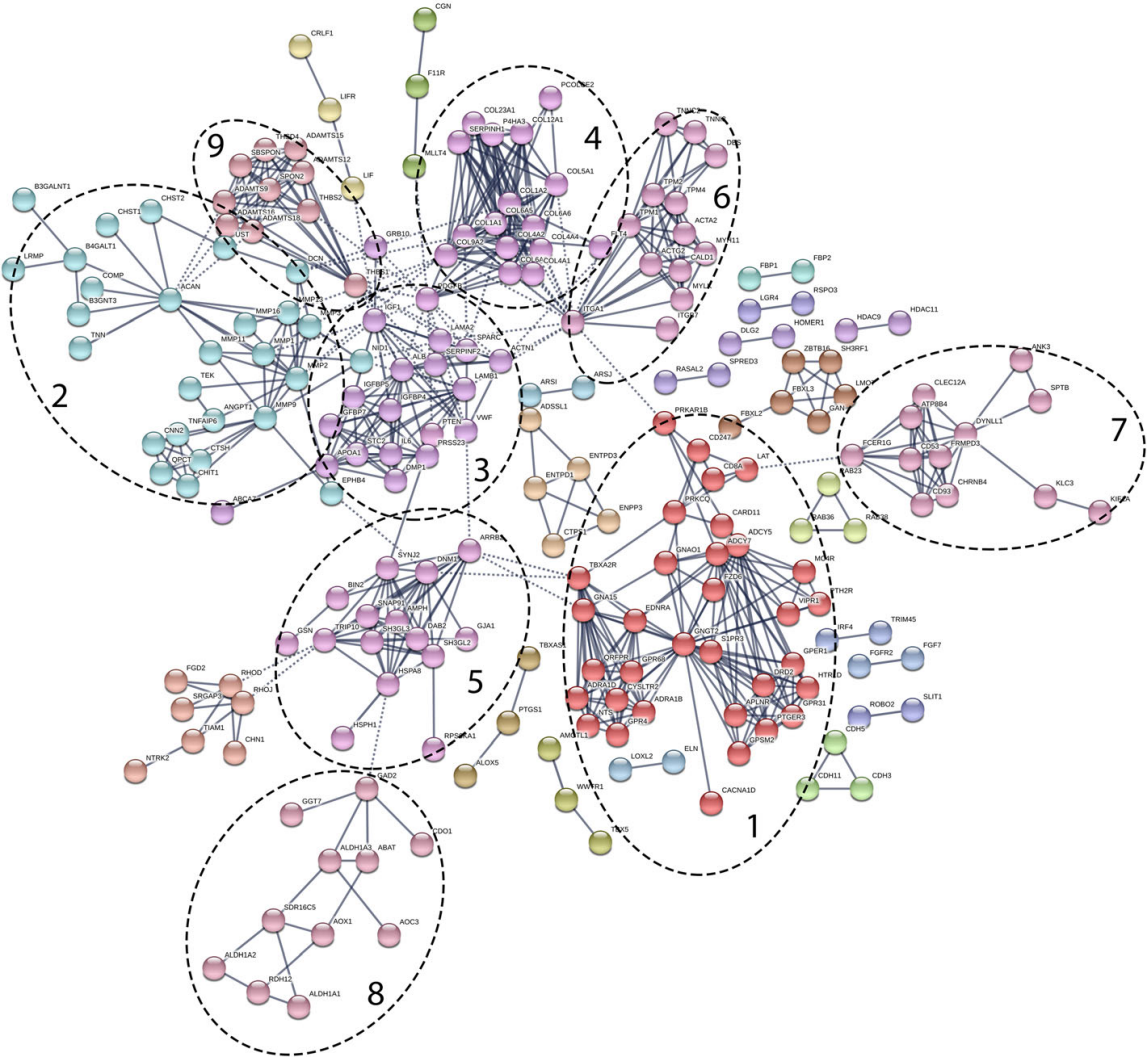
Clustering of DE genes using the MCL algorithm in STRING. Clusters with ten or more genes that were functionally annotated (Table 3) are circled and numbered. For clarity, DE genes that did not cluster with at least one other gene are not shown

Of the 762 DE genes, 213 were assigned to one of 28 unique clusters by STRING using the Markov clustering algorithm (Additional file 6). Nine of these clusters included ten or more genes (Fig. 4). The two largest clusters contained enough genes (33 and 27, respectively) to allow GO term enrichment testing in PANTHER. Cluster 1 was significantly enriched for biological process terms related to endothelial regulation (cell signaling, regulation of blood pressure and smooth muscle contraction), while Cluster 2 was enriched for biological process terms related to extracellular matrix organization and aminoglycan metabolic processes. Functional annotation of the other seven major clusters using PANTHER revealed functions related to cellular metabolism and homeostasis, vesicle formation and transport, and angiogenesis. Cluster composition and functional annotation is shown in Table 3.

**Table 3:** Clusters identified in STRING among DE genes

Cluster	Genes	Functional Annotation
1	TBXA2R, FZD6, GPER1, GPSM2, CD247, CACNA1D, PTH2R, GPR68, S1PR3, ADRA1B, CYSLTR2, QRFPR, NTS, GNA15, CD8A, PRKAR1B, ADCY5, MC4R, GPR31, PTGER3, GNGT2, GPR4, LAT, DRD2, ADCY7, HTR1D, ADRA1D, VIPR1, EDNRA, CARD11, APLNR, PRKCQ, GNAO1	Smooth muscle contraction, blood pressure regulation, cell signaling
2	ANGPT1, B3GNT3, COMP, UST, EPHB4, LRMP, MMP9, B4GALT1, CHST1, MMP11, TNFAIP6, CHIT1, CHST2, TNN, TEK, MMP2, ACAN, DCN, MMP16, B3GALNT1, NID1, CNN2, MMP13, CTSH, QPCT, MMP1, MMP3	Extracellular matrix organization, aminoglycan metabolism
3	IGF1, IGFBP7, PRSS23, IGFBP5, ALB, ABCA7, VWF, DMP1, SERPINF2, IL6, IGFBP4, SPARC, GRB10, LAMB1, LAMA2, ACTN1, STC2, APOA1	Protein and growth factor binding, signaling and structural receptors
4	P4HA3, COL4A1, COL5A1, COL1A2, COL6A6, COL1A1, COL6A3, COL23A1, COL9A2, PCOLCE2, COL6A5, FLT4, COL12A1, PDGFB, SERPINH1, COL4A4, COL4A2	Structural growth and development, collagen and extracellular matrix organization
5	GSN, SNAP91, SH3GL3, SH3GL2, BIN2, SYNJ2, PTEN, ARRB2, DAB2, TRIP10, GJA1, HSPH1, AMPH, HSPA8, RPS6KA1, DNM1	Vesicle formation and transport, response to cell stimulus
6	DES, MYLK, ACTA2, ITGB7, TNNC2, CALD1, TNNI3, TPM1, ACTG2, MYH11, TPM2, ITGA1, TPM4	Cytoskeletal organization
7	CLEC12A, FCER1G, CD93, DYNLL1, ANK3, CD53, CHRNB4, KIF1A, FRMPD3, KLC3, SPTB, ATP8B4	Signal transduction and cellular component localization
8	RDH12, ALDH1A3, CDO1, AOC3, ALDH1A1, GAD2, ABAT, ALDH1A2, AOX1, GGT7, SDR16C5	Cellular metabolic processes and response to cell stimulus
9	ADAMTS18, ADAMTS15, ADAMTS12, SBSPON, THBS2, ADAMTS9, SPON2, THBS1, ADAMTS16, THSD4	Angiogenesis

Functional annotation determined by GO-Slim Biological Process terms in PANTHER. Cluster numbers correspond to those shown in Fig. 4. A complete list of annotated clusters can be found in Additional file 6

## Discussion

Here we report differential expression of genes in an equine model of early PTOA. There was a clear response in synovial gene expression after disease induction when compared to healthy synovium (both “preOA” and from sham-operated joints). Within the 762 DE genes, overrepresented biological functions included those related to angiogenesis, extracellular matrix organization, and cell signaling. These functions were reflected in enriched pathways, which included those involved in extracellular matrix turnover, O-glycosylation of TSR domain-containing proteins, and growth factor signaling. Similarly, network and clustering analysis revealed major clusters of DE genes involved in extracellular matrix organization, endothelial regulation, synaptic vesicle formation and transport, and cellular metabolism and homeostasis. When considered together, these findings suggest both expected catabolic activity and efforts at healing and restoring homeostasis by the synovium in early PTOA.

These results are in contrast to previously reported gene expression data derived from end-stage disease. Genes previously reported to be upregulated in late-stage OA tissues (synovium, cartilage, and subchondral bone) were enriched for pathways and processes related to proteolysis, extracellular matrix disassembly, and collagen catabolism, while downregulated genes were enriched for pathways and processes related to cell proliferation and cellular response to stimuli [9, 10, 17–22]. These differences may be due to the fact that late-stage disease is dominated by gross tissue remodeling and chronic inflammation, while early disease is more likely to reflect initial responses to injury.

There are relatively few reports examining synovial gene expression in naturally-occurring OA in any species. Lambert et al. performed gene expression profiling of synovial biopsy samples from human patients undergoing knee replacement [21]. When synovium graded as grossly normal or reactive was compared to that graded as inflamed, genes falling within pathways related to inflammation, ECM catabolism, and angiogenesis were upregulated, while those related to anabolism were downregulated. Only 12 of the 58 genes reported differentially expressed in that study were also differentially expressed in our samples. However, notably, several were regulated in opposite directions when compared to our results, including ECM structural components COL1A2, COL6A3, and ACAN (downregulated in chronically inflamed synovium, but upregulated in our OA samples) and inflammatory enzymes ALOX5 and TBXAS1 (upregulated in chronically inflamed synovium, but downregulated in our OA samples). More recently, Zhu et al. used a co-expression network approach to identify thirteen “hub genes” among genes differentially expressed in human OA synovium when compared to normal tissue [23]. These genes fell within pathways related to autophagy, apoptosis, phosphorylation, and inflammation, and none of them were found to be differentially expressed in our samples. The lack of consistency between our findings and those of these previous studies could be attributed to differences in methodology (microarray versus RNAseq) or species (human versus equine), but could reflect actual biological differences in disease stage, as both of these previous studies were performed on tissue taken from joints with chronic OA at the time of joint replacement.

The MMP family, as well as the related ADAMTS protein family and other matrix-degrading enzymes, have long been associated with the onset and progression of OA [9], although evidence suggests that some of these proteins, including MMP-1, − 9, and − 13, may also play a role in postnatal growth and development [24]. MMP-13, in particular, is thought to play a central role in early OA [25]. MMP-13 was found to be upregulated in cartilage from early naturally-occurring OA in humans [18] as well as in cartilage and subchondral bone of rats as early as 1 week post-surgery in induced OA models [26]. We found MMP-13 to be strongly upregulated in our OA samples when compared to non-affected samples, along with MMP-1 and MMP-9. Other MMPs and ADAMTS were more modestly upregulated in the OA samples. Interestingly, expression of MMP-13 in chondrocytes has recently been shown to be regulated by the Notch signaling pathway via Runx2 [27], which is an essential transcription factor for chondrocyte maturation. In our samples, RUNX2 had a 1.8-fold higher expression in the OA samples compared to the non-affected samples. Further, our DE gene set was enriched for a RUNX2 regulatory pathway.

It should be acknowledged that although MMPs are considered key players in the pathology of OA primarily due to their catabolic effects on ECM components, they also play an important role in normal tissue homeostasis, as well as angiogenesis and wound healing [28]. Thus, the ratio of these molecules to their tissue inhibitors (TIMPs) may be more indicative of the balance between catabolism and anabolism than the absolute expression of MMPs [28]. TIMP expression has been reported in synovium [9], but was not differentially expressed in our samples. Although expression of TIMP was not elevated, evidence of anabolic activity in our OA samples was supported by enrichment in our DE gene set of an insulin-like growth factor (IGF-1) regulatory pathway, with IGF-1 and several of its binding proteins upregulated in our OA samples compared to unaffected tissue. IGF-1 is considered a key player in supporting cartilage growth and development, including enhancement of ECM synthesis [29].

A unique aspect of the equine model of PTOA used in the present study is that it does not cause substantial biomechanical instability of the joint, thus resulting in mild pathology. This is in contrast to many commonly used animal models across species, which destabilize the stifle via transection of the cruciate ligament and/or medial meniscus. Joint destabilization results in a rapid onset and progression of disease. For example, in mouse models utilizing anterior cruciate ligament transection (ACLT) alone or in combination with partial resection of the medial meniscus, both marked gene expression changes in articular cartilage and subchondral bone and histologic evidence of cartilage surface damage and proteoglycan loss were noted only 1 week after surgery, and by 6 weeks post-operatively significant macroscopic damage was evident [26, 30]. This short time course is useful from a research perspective, but does not accurately reflect clinical disease. It is of note that our synovial gene expression findings at 16 weeks post-operatively were very similar to those reported recently by Ayturk et al. in a minipig ACLT model 5–14 days post-operatively [31]. These similarities include upregulation of aggrecan, MMP 1 and 9, ADAMTS16, and cartilage oligomeric matrix protein (COMP) and downregulation of the fibronectin-binding protein myocilin and the ECM protein vitrin. A slower course of disease onset and progression may be beneficial when evaluating novel diagnostics and therapeutic interventions for early PTOA.

Our study has a few limitations. There was a relatively small number of samples, particularly in the preOA group in which some samples were lost. However, this was offset by the availability of paired sham samples from all individuals, which allowed creation of a larger “non-affected” sample set that was used in all analyses, and the use of each horse as its own control. Another limitation is that our gene expression data captures only a single time point in the course of disease post-injury. Ideally, samples would be collected at multiple points to demonstrate changes over time and to determine whether (and when) the catabolic response to injury has overwhelmed the tissue’s anabolic response. The ability to perform repeated sampling from the same individual is a major advantage of this equine model and could be done in future work. Paired cartilage biopsies could also be collected in the region of the injury to confirm that gene expression changes in synovium accurately reflect global joint health rather than tissue-specific inflammation, although this supposition is already supported by work showing correlation between expression of MMPs and ADAMTS in synovium and cartilage from human patients with naturally-occurring OA [9]. Finally, there are inherent limitations in our approach of functional annotation and pathway/network analysis. Gene ontology and pathway assignments are often based on textmining, which limits annotation to published information about gene-gene interactions, and may miss novel interactions. We attempted to address this limitation by eliminating textmining alone as a source of information for our STRING network analysis, but still may have missed biologically relevant interactions. Further, many DE genes fall within multiple functional annotation terms and pathways, which can complicate interpretation of these findings. It is possible that some of our overrepresented GO terms and enriched pathways were identified by chance, although our findings are consistent with other published literature in animal models of OA. Validation of tissue gene expression via quantitative polymerase chain reaction (qPCR) in an independent sample set as well as mechanistic investigations into the roles of differentially expressed genes in the pathogenesis of disease would be important next steps towards establishing the impact of our findings.

## Conclusions

Differential gene expression analysis in an equine osteochondral fragment model of PTOA revealed numerous pathways that may be involved in the onset and early progression of disease, reflecting a state of high metabolic activity and tissue turnover. These include expected pathways related to ECM organization, but also angiogenesis and growth factor signaling. This suggests that the synovium may retain the capacity to support healing and homeostasis in early disease, a finding supported by other recent work in a large animal model [31], although further validation to confirm these findings will be an important next step. This model may be a valuable tool in the investigation of novel diagnostic and therapeutic interventions for the early stages of this economically important disease.

## Methods

### Animal model

This work used banked synovial tissue samples that were collected from 11 adult horses during the course of a previously published study (live animal work for that previous study was carried out with approval from the Institutional Animal Care and Use Committee at the University of Minnesota, protocol #1002A78195) [16]. As described in that study, the experimental cohort was comprised of six female and five castrated male Quarter Horses (mean age of 5.6 ± 1.6 years), all of which were adopted out according to University protocol at the end of the study period. The model has previously been described [16]; however, briefly, an osteochondral fragment was created in one randomly chosen MCPJ at the proximal dorsomedial aspect of the first phalanx. The fragment was replaced in the fragment bed after creation so that subchondral bone was not exposed to the opposing cartilage surface. The opposite MCPJ was sham-operated. After a 2 week recovery period, the horses were treadmill-exercised for 14 weeks and then the osteochondral fragment was removed (16 weeks after creation). Synovial tissue samples were collected arthroscopically from the MCPJ before (preOA) and 16 weeks after (OA) experimental induction of OA as well as from the sham-operated joints (sham) [16]. Specifically, synovium was collected from the dorsomedial and dorsolateral aspects of the MCPJ approximately 1 cm proximal to the medial/lateral dorsoproximal eminences of the first phalanx. Synovium samples were placed in RNAlater (Qiagen, Valencia, CA) and stored at − 80 °C until further processing.

Clinical, radiographic, histologic, and arthroscopic characterization of the disease induced by this MCPJ osteochondral fragment model has been extensively described in the previously published work by Boyce et al. [16]. However, a summary of these findings is relevant to help contextualize the results of the current study. Injured (OA) joints had higher effusion scores than did preOA or sham joints (which were not different from each other). A transient lameness was observed in OA joints that resolved by 16 weeks, although a positive response to MCPJ flexion was persistent. Total radiographic scores and enthesiophyte scores were significantly higher in OA joints than preOA or sham joints (which were not different from each other), although radiographic changes were subjectively mild. Athroscopic scores were higher in OA joints than in preOA or sham joints (which were not different from each other), and this was primarily attributable to the presence of cartilage wear lines opposite the osteochondral fragment. Total histologic scores for synovium were higher in the OA joints than in preOA or sham joints (which were not different from each other), and this was primarily attributable to an increase in vascularity. Collectively, these changes were consistent with early PTOA.

### RNA extraction and sequencing

Frozen samples were crushed to powder with a mortar and pestle and placed in tubes containing ceramic beads (Precellys® 2 ml Hard Tissue Homogenizing Ceramic Beads CK28, Cayman Chemical, Ann Arbor, MI) and TRIzol reagent (Invitrogen, Carlsbad, CA). Mechanical homogenization was performed for cell lysis (Precellys® Minilys, Bertin Corp., Rockville, MD) prior to RNA extraction on spin columns using the RNeasy Micro Kit (Qiagen, Valencia, CA) per manufacturer instructions. RNA concentration was measured using a NanoDrop 2000 spectrophotometer (ThermoFisher Scientific, Waltham, MA), with 260/280 absorbance ratio evaluated for evidence of contamination. Five preOA samples did not have RNA of sufficient quantity and quality for sequencing and were lost to further analysis, resulting in a total of 28 samples. These samples were checked for RNA quality number (RQN) on an AATI Fragment Analyzer (Advanced Analytical Technologies, Inc., Ames, IA) (Additional file 1). RNAseq libraries were prepared with the TruSeq Stranded mRNAseq Sample Prep Kit (Illumina, San Diego, CA). The libraries were quantitated by qPCR and sequenced (100 base-pair, paired-end) on three lanes for 101 cycles from each end of the fragments on an Illumina HiSeq 4000 using a HiSeq 4000 sequencing kit version 1 (Illumina, San Diego, CA) at the University of Illinois Roy J Carver Biotechnology Center. Fastq files were generated and demultiplexed with the bcl2fastq v2.20 Conversion Software (Illumina, San Diego, CA). RNA sequences have been deposited into the National Center for Biotechnology Information (NCBI) Gene Expression Omnibus (GSE144031).

### Data analysis

#### Alignment, gene level quantification, and surrogate variable analysis

Sequence reads were quality checked using fastQC (Barbraham Bioinformatics, Cambridge, UK; www.bioinformatics.barbraham.ac.uk/projects/fastqc/), then adaptors were trimmed with Trimmomatic [32], and the results inspected with multiQC [33]. Quasi-mapping to NCBI’s EquCab3.0 reference genome (Annotation Release 103) was performed with Salmon [34] (version 0.11.3) with the --validateMappings, --rangeFactorizationBins 4, --gcbias, and --seqbias options enabled. Transcript aggregation at the gene level was performed with tximport [35].

Of the 29,196 genes identified in EquCab3.0, 15,177 genes were expressed in the synovial tissue at a level of at least 1 count per million (cpm) in six samples, which corresponded to the smallest group of samples (preOA). These gene counts were retained, while genes with lower abundance were filtered out. TMM normalization was performed for these 15,177 genes with edgeR [36] using the ‘cpm’ function (prior.count = 3) on log_2_-based counts per million (logCPM) transformed count values (Additional file 7). In order to account for unmodeled confounders, surrogate variable analysis (SVA) [37–39] was performed utilizing the R package sva [40]. SVA allows removal of unwanted sources of biologic or technical variation while protecting the contrasts due to the primary variable of interest in the model (in this case, OA versus non-affected samples). This estimated eight continuous quantitative variables that were utilized in downstream differential expression analysis as covariates. The surrogate variable effects were also removed from normalized logCPM values for the purpose of visualization (Additional file 8).

#### Differential expression testing

The difference in library sizes between the smallest and largest was approximately 2-fold, so the limma-trend method [41, 42] was selected to robustly model differential expression (DE) comparing the three treatment groups (preOA, OA, sham) plus the eight surrogate variables. Limma-trend is similar to the more popular limma-voom but performs as well or better than limma-voom if library sizes are less than three-fold different between samples [41]. Further ‘treat’ testing [43], implemented within the limma package, was performed in order to simultaneously test for significance relative to a biologically meaningful threshold, set at log_2_(1.1)-fold change. The false discovery rate (FDR) [44] was calculated to correct for multiple testing using a significance level of q < 0.05.

Initially, three groups of interest were identified: preOA, OA, and sham. DE analysis as described revealed no DE genes between the preOA and sham groups. Therefore, the analysis was repeated by combining the preOA and sham samples, then comparing the 17 samples now designated “non-affected” to the 11 affected OA samples.

#### Functional annotation and pathway analysis

Annotation information (gene symbol, gene name, and Entrez gene ID) for each gene was collected using Bioconductor’s [45] AnnotationHub [46] web resource. Entrez gene IDs were converted to FASTA protein sequences using NCBI’s “Batch Entrez” web resource (https://www.ncbi.nlm.nih.gov/sites/batchentrez), then input into the eggNOG database [47] to find consensus orthologues across species, particularly for EquCab3.0 genes that have not yet been manually curated by NCBI. UniProt gene IDs could then be assigned for further functional annotation analysis.

DE genes were input into PANTHER [48] (version 14.1) for gene ontogeny (GO) assignment. The PANTHER Overrepresentation Test was performed to identify enriched GO-Slim terms for molecular function, biological process, and cellular component within the gene set [49]. For all tests, a Fisher’s exact test with FDR correction was used, with significance set at FDR *p* < 0.05.

Pathway analysis of DE genes was performed using the Reactome Pathway Knowledgebase [50]. This program employs a hypergeometric test, correcting the resultant probability score for FDR, for which significance was set at p < 0.05. As an alternative approach to network analysis, STRING v11 [51] was used to perform gene clustering with a Markov clustering (MCL) algorithm with a minimum required interaction score of 0.9 and the inflation factor set at 1.4. Clusters containing at least 10 genes were functionally annotated with GO terms using PANTHER.

## Supplementary Information

**Additional file 1.** RNA quality number (RQN) and number of reads for all samples.

**Additional file 2.** Genes differentially expressed with a fold-change (FC) cut-off > |1.1|. Genes upregulated in OA samples are represented by a positive FC; genes downregulated in OA samples are represented by a negative FC.

**Additional file 3.** Hierarchical listing of overrepresented gene ontology (GO) terms. Different tabs in the spreadsheet correspond to the categories of Biological Process, Molecular Function, and Cellular Component.

**Additional file 4.** Graphical representations of the terminal hierarchical overrepresented GO terms from Table 1. Pie charts are based on the proportion of genes in the analyzed DE list (comparing OA to non-affected samples) falling within the each of the terms shown. The number of genes included in each overrepresented GO term is listed next to the respective term.

**Additional file 5.** Genes falling within pathways identified as enriched by Reactome analysis.

**Additional file 6.** DE genes assigned to clusters by STRING using a MCL algorithm. Functional annotation of clusters with 10 or more genes was performed based on GO-Biological Process ontology in PANTHER.

**Additional file 7.** Post-filtering TMM normalization factors to correct for RNA composition.

**Additional file 8.** Multidimensional scaling (MDS) on the top 5000 most variable genes before (A) and after (B) removal of surrogate variables.

## Data Availability

All data generated during the current study are available in the National Center for Biotechnology Information (NCBI) Gene Expression Omnibus (GSE144031).

## Abbreviations

ACLT: Anterior cruciate ligament transection
ADAMTS: A disintegrin and metalloproteinase with thrombospondin motifs
CPM: Counts per million
DE: Differential expression/differentially expressed
ECM: Extracellular matrix
FC: Fold-change
FDR: False discovery rate
GO: Gene ontology
IGF: Insulin-like growth factor
MCL: Markov clustering
MDS: Multi-dimensional scaling
MCPJ: Metacarpophalangeal joint
MMP: Matrix metalloproteinase
NCBI: National Center for Biotechnology Information
OA: Osteoarthritis; in the context of this work, refers to samples collected after experimental induction of disease; similarly, preOA refers to samples collected before experimental induction of disease
PTOA: Post-traumatic osteoarthritis
qPCR: Quantitative polymerase chain reaction
RNA: Ribonucleic acid
TIMP: Tissue inhibitor of metalloproteinase
