# Supplementary material for: Differential gene expression analysis reveals pathways important in early post-traumatic osteoarthritis in an equine model
Source: BMC Genomics. 2020 Nov 30;21:843. doi: 10.1186/s12864-020-07228-z (PMC7708211; doi:10.1186/s12864-020-07228-z)
Supplement: Supplementary file 1 — Additional file 1. RNA quality number (RQN) and number of reads for all samples. [file 12864_2020_7228_MOESM1_ESM.docx]

**Additional file 1:** RNA quality number (RQN) and number of reads for all samples.

| **Sample** | **Category** | **RNA Quality Number**  **(RQN)** | **Number of Reads** |
| --- | --- | --- | --- |
| 1 | sham | 8.2 | 26,705,099 |
| 2 | preOA | 8.9 | 17,228,646 |
| 3 | OA | 6.0 | 16,195,344 |
| 4 | preOA | 8.5 | 22,210,893 |
| 5 | preOA | 8.0 | 23,173,840 |
| 6 | preOA | 8.1 | 17,506,836 |
| 7 | OA | 7.5 | 21,590,263 |
| 8 | preOA | 8.1 | 22,236,150 |
| 9 | preOA | 8.3 | 20,545,508 |
| 10 | sham | 7.9 | 22,559,753 |
| 11 | OA | 7.3 | 23,233,560 |
| 12 | OA | 7.3 | 15,933,687 |
| 13 | OA | 6.4 | 24,924,463 |
| 14 | sham | 6.6 | 15,694,658 |
| 15 | OA | 6.3 | 21,532,611 |
| 16 | sham | 8.9 | 16,234,902 |
| 17 | sham | 8.1 | 24,240,218 |
| 18 | sham | 8.5 | 26,348,728 |
| 19 | OA | 7.6 | 26,020,713 |
| 20 | OA | 7.2 | 24,622,849 |
| 21 | sham | 7.9 | 16,554,140 |
| 22 | OA | 7.6 | 19,971,843 |
| 23 | sham | 7.9 | 17,148,271 |
| 24 | sham | 8.5 | 21,703,971 |
| 25 | OA | 8.5 | 25,129,550 |
| 26 | sham | 8.1 | 20,883,840 |
| 27 | sham | 7.9 | 29,449,660 |
| 28 | OA | 7.6 | 25,765,733 |
