# Supplementary material for: Differential gene expression analysis reveals pathways important in early post-traumatic osteoarthritis in an equine model
Source: BMC Genomics. 2020 Nov 30;21:843. doi: 10.1186/s12864-020-07228-z (PMC7708211; doi:10.1186/s12864-020-07228-z)
Supplement: Supplementary file 4 — Additional file 4. Graphical representations of the terminal hierarchical overrepresented GO terms from Table 1. Pie charts are based on the proportion of genes in the analyzed DE list (comparing OA to non-affected samples) falling within the each of the terms shown. The number of genes included in each overrepresented GO term is listed next to the respective term. [file 12864_2020_7228_MOESM4_ESM.docx]

**Additional file 4:** Graphical representations of the terminal hierarchical overrepresented GO terms from **Table 1**. Pie charts are based on the proportion of genes in the analyzed DE list (comparing OA to non-affected samples) falling within each of the terms shown. The number of genes included in each overrepresented GO term is listed next to the respective term.
