# Supplementary figures and images for: Differential gene expression analysis reveals pathways important in early post-traumatic osteoarthritis in an equine model
Source: BMC Genomics. 2020 Nov 30;21:843. doi: 10.1186/s12864-020-07228-z (PMC7708211; doi:10.1186/s12864-020-07228-z)

**Additional file 7:** Post-filtering TMM normalization factors to correct for RNA composition.


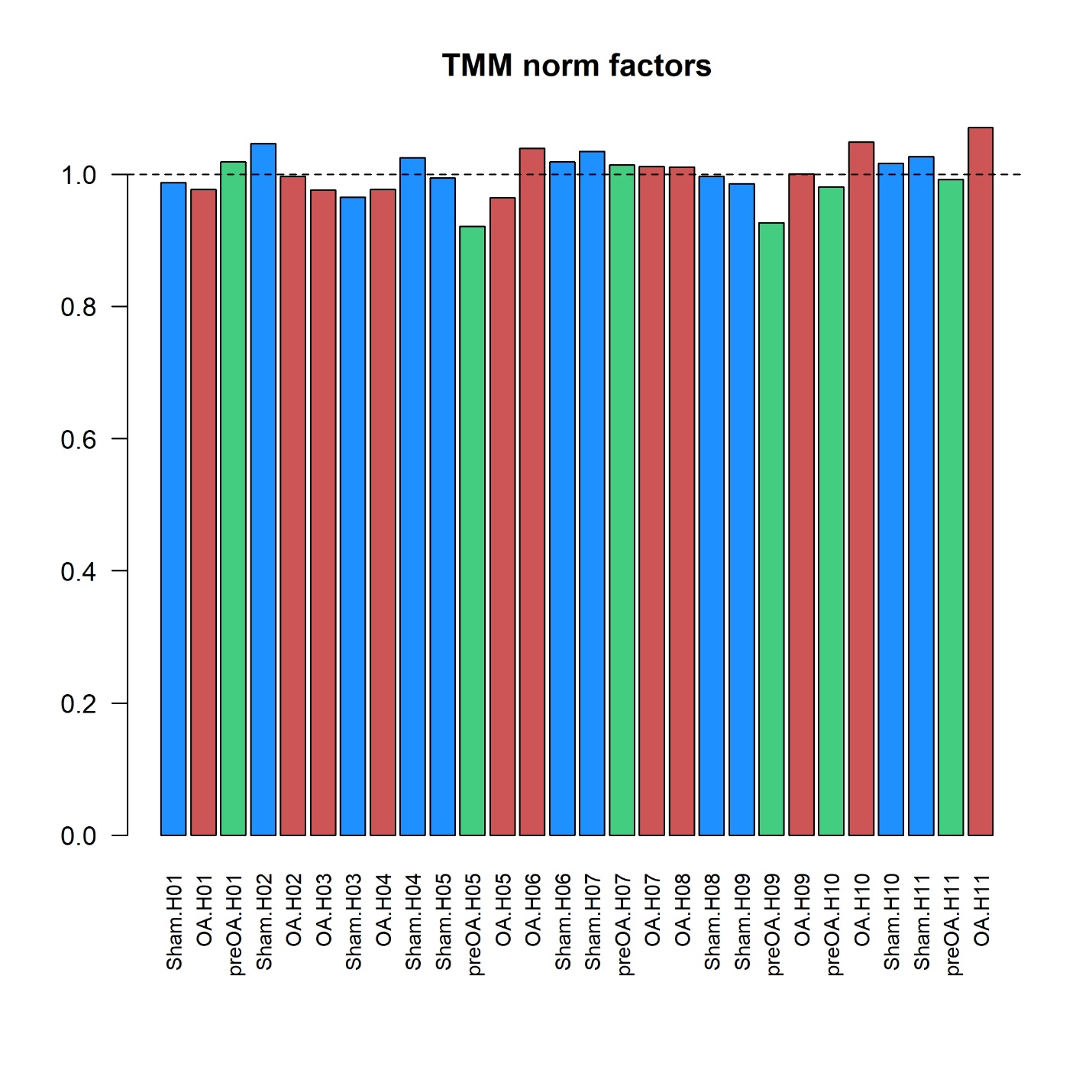

Supplement: Supplementary file 7 — Additional file 7. Post-filtering TMM normalization factors to correct for RNA composition. [file 12864_2020_7228_MOESM7_ESM.docx]
