# Supplementary material for: Differential gene expression analysis reveals pathways important in early post-traumatic osteoarthritis in an equine model
Source: BMC Genomics. 2020 Nov 30;21:843. doi: 10.1186/s12864-020-07228-z (PMC7708211; doi:10.1186/s12864-020-07228-z)

**Additional file 8:** Multidimensional scaling (MDS) on the top 5,000 most variable genes before (A) and after (B) removal of surrogate variables. (B) corresponds to **Figure 1** in the main text. Surrogate variable analysis allows removal of unwanted sources of biologic variation while protecting the contrasts due to the primary variable of interest in the model (in this case, affected versus unaffected samples).


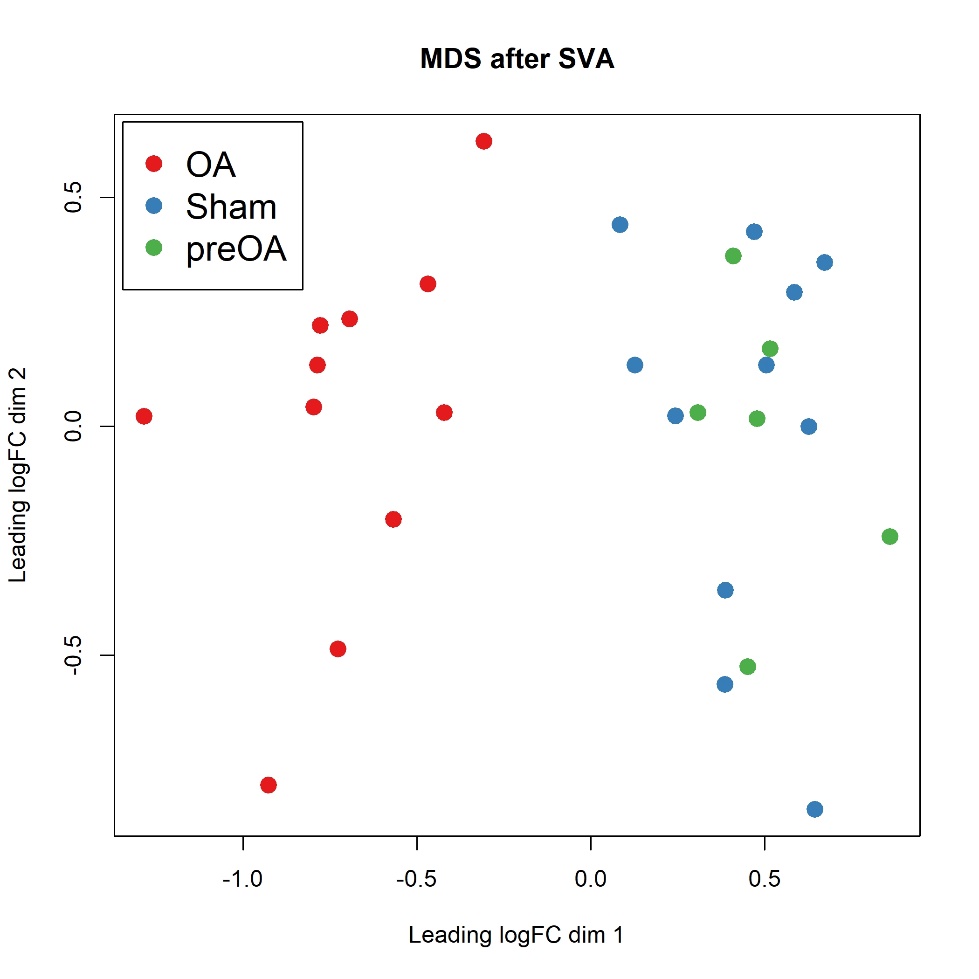


B

A


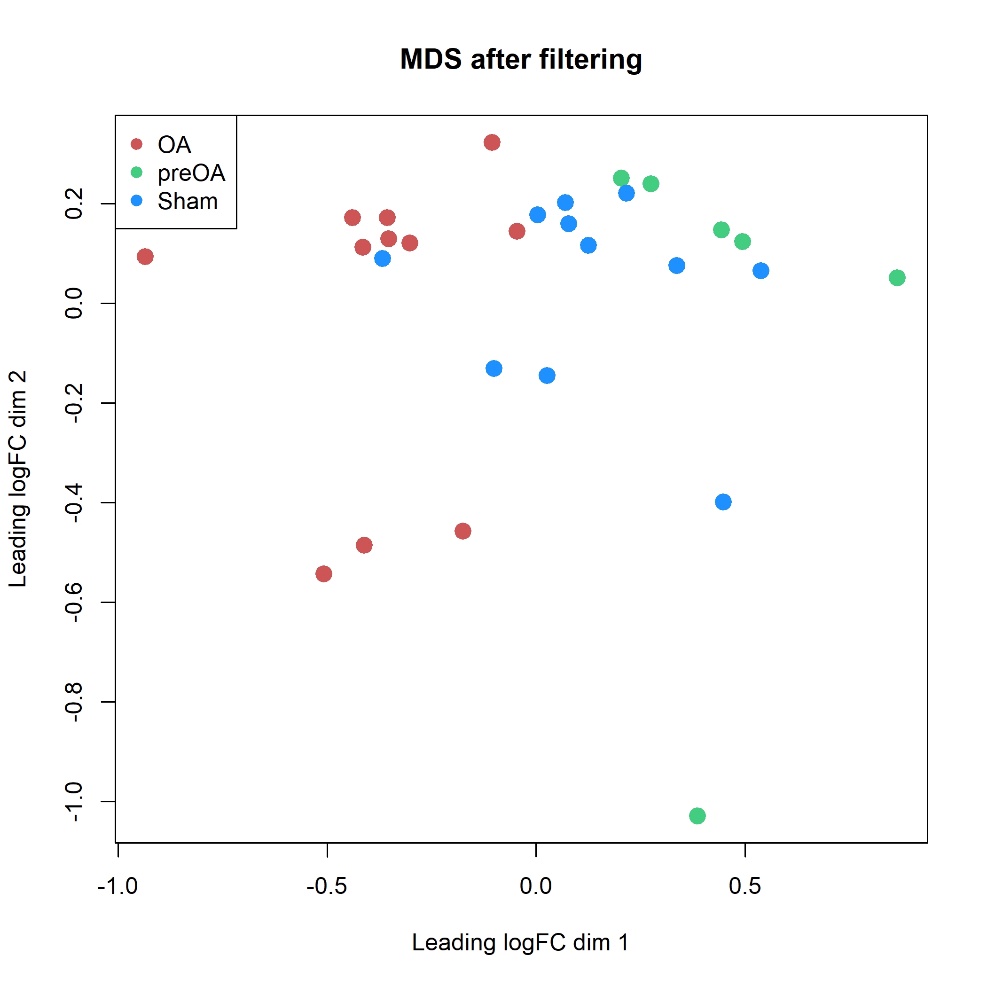

Supplement: Supplementary file 8 — Additional file 8. Multidimensional scaling (MDS) on the top 5000 most variable genes before (A) and after (B) removal of surrogate variables. [file 12864_2020_7228_MOESM8_ESM.docx]
